# Antibacterial Properties of an Experimental Dental Resin Loaded with Gold Nanoshells for Photothermal Therapy Applications

**DOI:** 10.3390/jfb15040100

**Published:** 2024-04-11

**Authors:** Shayan Darvish, Dana-Gabriela Budala, Ancuta Goriuc

**Affiliations:** 1 Department of Oral Health Sciences, Faculty of Dentistry, The University of British Columbia, Vancouver, BC V6T 1Z3, Canada; sdarvish@umich.edu; 2 Department of Prosthodontics, Faculty of Dental Medicine, “Grigore T. Popa” University of Medicine and Pharmacy, 16 Universității Street, 700115 Iași, Romania; 3 Department of Biochemistry, Faculty of Dental Medicine, “Grigore T. Popa” University of Medicine and Pharmacy, 16 Universității Street, 700115 Iași, Romania; ancuta.goriuc@umfiasi.ro

**Keywords:** dental caries, gold, nanoshells, photothermal therapy, dental resin, nanoparticles, *Streptococcus mutans*

## Abstract

This study explored the chemical and antibacterial properties of a dental resin loaded with gold nanoshells (AuNPs) in conjunction with photothermal therapy (PTT) as a novel method against *Streptococcus mutans* (*S. mutans*) to prevent secondary caries. First, a 20-h minimum inhibitory concentration (MIC) assay was performed on solutions of AuNPs with planktonic *S. mutans* under an LED device and laser at 660 nm. Next, resin blends containing 0, 1 × 10^10^, or 2 × 10^10^ AuNPs/mL were fabricated, and the degree of conversion (*DC*) was measured using an FTIR spectroscopy. Lastly, a colony forming unit (CFU) count was performed following 24 h growth of *S. mutans* on 6 mm diameter resin disks with different light treatments of an LED device and a laser at 660 nm. The MIC results only showed a reduction in *S. mutans* at AuNP concentrations less than 3.12 µg/mL under a laser illumination level of 95.5 J/cm^2^ compared to the dark treatment (*p* < 0.010 for each). CFU and *DC* results showed no significant dependence on any light treatment studied. The AuNPs expressed antibacterial effects following PPT against planktonic S. mutans but not in a polymerized dental adhesive resin. Future studies should focus on different shapes, structure, and concentrations of AuNPs loaded in a resin blend.

## 1. Introduction

Dental caries remain one of the most common diseases in the world [1]. With an ever-increasing global population and increased life expectancy of individuals, there is a significant need to develop more appropriate approaches to address dental caries [2]. Due to the esthetic that resin-based materials provide, they are more appealing than amalgam; however, it is reported that up to 70% of the restorations currently placed in the clinic are to replace previously failed ones [3,4]. The foremost reason for this failure is secondary caries. The gap formation occurring during polymerization shrinkage in resin-based materials, degradation of the hybrid layer over time, and biofilm formation in the gap are leading factors for microleakages and can result in carious lesions [3,5,6]. A new approach that endeavors to instill dental resin blends with antibacterial properties through the use of nanoparticles against *S. mutans* as one type of predominant cariogenic bacteria [7] could make significant advancements in dental caries management. It has been concluded in a recent review study by Chen et al. (2020) that novel anti-caries materials and nanoparticles should be developed with high efficiency to overcome dental caries [8]. To date, nanoparticles such as copper [9], TiO_2_ [10], zinc oxide [11], and silver [12] have been studied as an additive into dental materials including adhesives, glass ionomers, and composite resins. Moreover, the addition of photosensitive nanoparticles such as zinc oxide [13] and other compounds such as curcumin [14] and riboflavin (B2) [15] has been shown to maintain resin antibacterial properties following ageing for 28 days and an application of photodynamic therapy (PDT). However, more investigation is needed to improve the antibacterial properties of freshly prepared resin upon loading of the additives for PDT.

Another novel approach which has been of increasing interest in the field of dentistry is photothermal therapy (PTT), where the light energy is transferred to heat as a result of the electromagnetic fields of the photothermal agents (PTTAs) present [16]. PTT has shown promising results against various microbiological species, including *S. mutans* [17,18,19,20]. Additionally, PTT has been successful in the treatment of cancerous cells by using near-infrared wavelength light, which is more permeable for reaching tumors under the skin and is a less toxic treatment than traditional chemotherapy [18]. Currently, different PTTAs exist and are under investigation for PTT. These include noble metal nanoparticles, polymers, organic materials, and oxides. An ideal PTTA should have minimal toxicity, have high efficiency in converting light to heat, be durable, and be economic [16,18].

Noble metal nanoparticles such as silver, gold, copper, and platinum can be a suitable choice for PTT due to their surface plasmon resonance (SPR), which helps in converting applied light to heat energy [21,22]. Gold nanoparticles (AuNPs) are particularly appealing for PTT due to their stability and lowest measured mammalian cell toxicity compared to other metallic nanoparticles suitable for PTT application [18,19,23,24,25]. Additionally, due to the strong SPR of AuNPs, they have previously been found to be efficacious for PTT [23,25,26]. *S. mutans* has been reported to be permanently deactivated at 55 °C and die at temperatures greater than 60 °C [27,28]. Thus, a temperature difference of 18 °C to 23 °C from the initial oral cavity temperature (37 °C) is expected to be adequate to eliminate *S. mutans* and stop acid production.

Gold nanoshells have been shown to be more efficient in raising the temperature compared to gold nanorods [29,30]. Therefore, by using nanoshells, fewer particles are needed to increase the local temperature than would be required in the nanorod form [31,32]. Owing to the results of prior studies, AuNPs in nanoshell form, and with a silica core, were chosen as the PTTA to be investigated in this study.

To date, no study has explored the potential benefit of combining the loading of AuNPs into a dental resin blend to benefit from their PTT effect on the antibacterial properties of the resin. The aim of this study was to assess the antibacterial effects of an experimental dental resin incorporated with gold nanoshells against *S. mutans* without compromising the polymerization process of the resin blend. The results of this study can pioneer the application of PTT to control oral pathogenic bacteria linked to dental caries, which could sustain antimicrobial properties over time, and eventually reduce the impact of the disease on the overall health of individuals, as well as its significant economic impact.

## 2. Materials and Methods

### 2.1. Resin Blend Preparation

An experimental resin blend was prepared in accordance with Table 1.

The first four reagents in Table 1 were mixed in order within an amber bottle on a magnetic stirring plate. The ethanol was added directly for the 0% AuNP (unloaded) blend. However, for the AuNP-containing resin blends, the AuNP was first added to the ethanol at the requisite concentration before transferring the mixture to the resin blend on the stirring plate. For the 0% AuNP blend, the BisEMA was sourced from either Scientific Polymer Products (“SPP”, Ontario, NY, USA) or Esstech Inc. (“EI”, Essington, PA, USA) due to product discontinuation by SPP. Meanwhile, for the 1 × 10^10^ AuNP/mL blend, the BisEMA was solely sourced from SPP, and for the 2 × 10^10^ AuNP/mL blend, the BisEMA was sourced from EI. TEEGDMA was purchased from SPP (Ontario, NY, USA), while CQ and amino were purchased from Sigma Aldrich, Inc. (St. Louis, MO, USA).

Lastly, gold nanoshells were purchased from NanoComposix (San Diego, CA, USA) at a concentration of 9.5 × 10^10^ particles/mL equal to 1 mg/mL in 0.02 mM potassium carbonate. They had a total size of 120 nm, with an 80 nm silica core and a 20 nm gold shell with carboxyl surface functionalization. A certificate of analysis of the gold nanoshells including characterization is available as a Supplementary Material. To replace the solvent with ethanol in preparation for their addition to the resin blend, the purchased solution of nanoparticles was first centrifuged at 5000 rpm for 5 min. The aqueous supernatant was replaced with ethanol to obtain the required AuNP concentration to match either 1 × 10^10^ AuNP/mL or 2 × 10^10^ AuNP/mL in the resin blend.

### 2.2. Degree of Conversion (DC)

A Fourier transform infrared (FTIR) spectrometer (Spectrum Two, Perkin Elmner, Shelton, CT, USA) was used to determine the *DC* of the blends as a function of the AuNP concentration. A drop of the resin blend was first applied between two transparent polyacetate films. Following a background reading of two clean polyacetate films, the sample spectra of the unpolymerized sample was read. Finally, 60 s of ~750 mW/cm^2^ light was then applied to the sample to encourage photopolymerization using a Valo^TM^ light curing unit (South Jordan, UT, USA). All spectra were collected from 400 cm^−1^ to 2000 cm^−1^ (36 readings; resolution of 4 cm^−1^). The peak absorbance for aliphatic and aromatic double carbon bonds was identified at 1636 cm^−1^ and 1608 cm^−1^, respectively. Equation (1) was used to calculate the *DC*.

(1) $$DC=100\%\times (1-[\frac{Light\;cured\;sample((abs\;1636\;{cm}^{-1})/(abs\;1608\;{cm}^{-1}))\;}{Noncured\;sample((abs\;1636\;{cm}^{-1})/(abs\;1608\;{cm}^{-1}))}])$$

### 2.3. Bacterial Strain and Growth Conditions

A standard strain of *Streptococcus mutans* (UA159) was purchased from the American Type Culture Collection (ATCC 700610; Rockville, MD, USA). Stock cultures were maintained at −80 °C, reactivated on 5% sheep blood agar plates (BBL, Becton, Dickinson and Company, Sparks, MD, USA), and incubated at 37 °C for 48 h. Next, the pre-inoculum was formed by transferring single colonies (10–12) to a tube containing 5 mL of brain–heart infusion (BHI) broth culture media (BD BBL, Becton, Dickinson and Company, Sparks, MD, USA) supplemented with 1% glucose. The pre-inoculum was kept overnight in an incubator at 5% CO_2_ and 37 °C (Isotemp CO_2_ incubator, Thermo Fisher Scientific, Marietta, OH, USA). The pre-inoculum was then read at an optical density (OD) of 600 nm using an Epoch microplate reader (BioTek, Winnoski, VT, USA) and diluted with BHI broth until the OD600 read 0.08–0.10 (corresponding to 1.5 × 10^8^ colony forming units (CFU)/mL). The inoculum was diluted further with BHI broth to obtain a final *S. mutans* concentration of 1 × 10^6^ CFU/mL.

### 2.4. Minimum Inhibitory Concentration (MIC)

A stock solution of 400 µg/mL of AuNPs was first prepared in sterile water before being serially diluted by a factor of 2 to 1.56 µg/mL using sterile water. Next, 100 µL of each AuNP concentration was added to a 96-well plate and 100 µL of 1 × 10^6^ CFU/mL planktonic *S. mutans* in BHI broth. Controls for this assay included 0 µg/mL AuNPs with *S. mutans*, and a 50:50 mixture of BHI–water without bacteria. Each treatment (Table 2) had its own plate, and this assay was performed in triplicate in each of 3 runs (n = 9). The wavelength of the LED and laser (power setting of 100 mW) was set at 660 nm to have the maximum photothermal effect possible for the gold nanoshells in our study.

After treatment, the plates were incubated for 20 h in an incubator at 5% CO_2_ and 37 °C (Isotemp CO_2_ incubator, Thermo Fisher Scientific, Marietta, OH, USA). The plates were then visually assessed for turbidity and the OD600 was read with an Epoch microplate spectrophotometer (BioTek, Winnoski, VT, USA).

### 2.5. Colony Forming Unit (CFU) Assay

For the unloaded resin blends and blends containing 1 × 10^10^ AuNP/mL, 6 mm diameter resin disks were fabricated using a 1 mm thick PVS mold and a 750 mW/cm^2^ Valo^TM^ light curing unit (South Jordan, UT, USA) applied for 60 s on both sides of the disk. Meanwhile, for the 2 × 10^10^ AuNP/mL concentration, the unloaded disks were first prepared as mentioned previously and a layer of coating blend containing 2 × 10^10^ AuNP/mL was applied with an additional 60 s of photopolymerization. All disks were then autoclaved for 30 min at 121 °C.

Next, *S. mutans* was then seeded on the surface of each disk by adding 0.5 mL the 1 × 10^6^ CFU/mL *S. mutans* with BHI into the wells of a 24-well plate and incubated for 24 h (5% CO_2_, 37 °C, Isotemp CO_2_ incubator, Thermo Fisher Scientific, Marietta, OH, USA). The disks were then washed with 1 mL of sterile phosphate-buffered saline (PBS) and subjected to a light treatment listed in Table 3. The wavelength of the LED and laser (power setting of 100 mW) was set at 660 nm to have the maximum photothermal effect possible for the gold nanoshells in our study. An IR thermometer (Fluke 62 MAX, Everett, WA, USA) was used to observe the temperature difference at the surface of the disk.

Following the light treatment, the disks were transferred to pre-labeled microcentrifuge tubes containing 1 mL of sterile PBS and sonicated for 30 s at amplitude 20 to detach *S. mutans* from the surface of the disks (QSonica sonicators, Newton, CT, USA). The disks were then removed, and a serial dilution was performed by a factor of 10 using sterile PBS until a final dilution of 10^−7^ was reached. A total of 10 μL of each dilution was then transferred onto blood agar plates and the plates were incubated for 48 h (5% CO_2_, 37 °C, Isotemp CO_2_ incubator, Thermo Fisher Scientific, Marietta, OH, USA). After incubation, the colonies were counted and the final CFU concentration was calculated using the following formula:

CFU/mL = [(# of colonies) × (1/dilution factor) × 1000 µL/mL)]/(Volume transferred to plates),(2)

This assay was performed in triplicate in each of the 3 runs (n = 9).

### 2.6. UV-Vis Analysis

A total of 100 µL of each resin blend concentration (0%, 1 × 10^10^ particles/mL, and 2 × 10^10^ particles/mL concentration) was added to a 96-well plate and the absorbance was read at 660 nm, which is the peak absorbance for the gold nanoshells used in this study using an Epoch microplate reader (BioTek, Winnoski, VT, USA). This test was performed to check the gold absorbance capability after their addition to the resin blends. The blends for EI and SPP were analyzed separately in comparison to the gold nanoshells added to them at 2 × 10^10^ AuNP/mL and 1 × 10^10^ AuNP/mL, respectively.

### 2.7. Scanning Electron Microscopy (SEM) and Energy Dispersive X-ray (EDX)

Three sample resin disks from every concentration (0%, 1 × 10^10^ particles/mL, and 2 × 10^10^ particles/mL) were glued to the 12.5 mm SEM stubs. The disks were made with a Mylar strip to have a smooth Mylar surface. The stubs were stored in a desiccator for a week to dry well. Each stub was carbon coated for conductivity (10–20 mm carbon coating). An SEM Zeiss Crossbeam 350 (Oberkochen, Germany) was used on multiple locations on each sample to capture the surface topography of the disks. The energy dispersive X-ray (EDX) analysis was performed on the disk surfaces to confirm the presence of gold nanoparticles on the surface of the resin disks with the same device.

### 2.8. Statistical Analysis

The statistical analysis included a multi-factor univariate general linear model and a post-hoc Tukey test using SPSS software (Version 28, IBM, Chicago, IL, USA). Statistical significance was accepted at *p* < 0.05.

## 3. Results

### 3.1. Degree of Conversion (DC)

**Figure 1:**
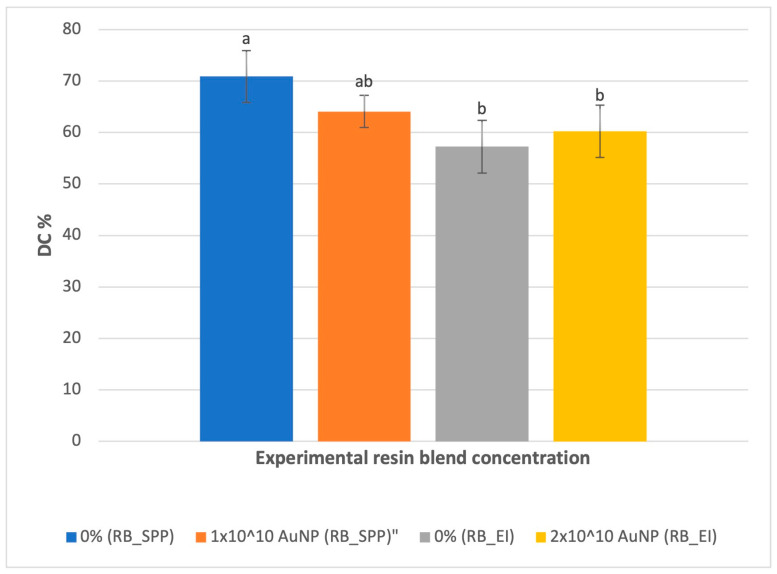
Degree of conversion for resin blends as a function of AuNP concentration. (Results are presented as mean ± one standard deviation (n = 6). Statistically different means are assigned different letters (*p* < 0.05)).

The degree of conversion (*DC*) was tested to determine whether photopolymerization showed any dependence on AuNP concentration. Interestingly, the source of BisEMA was found to have a significant impact on the *DC* of the unloaded blend (*p* < 0.001). As such, the remaining results are separated according to the BisEMA source–Scientific Polymer Products (SPP) or Esstech Inc. (EI). In each instance, the unloaded blend (0% AuNP) serves as a sample control. FTIR analysis confirmed that the addition of gold nanoshells to the resin blend did not affect the *DC* when compared to the 0% blend (*p* = 0.087 for RB_SPP and 0.685 for RB_EI) (Table 4) (Figure 1).

### 3.2. Minimum Inhibitory Concentration (MIC) Assay

**Figure 2:**
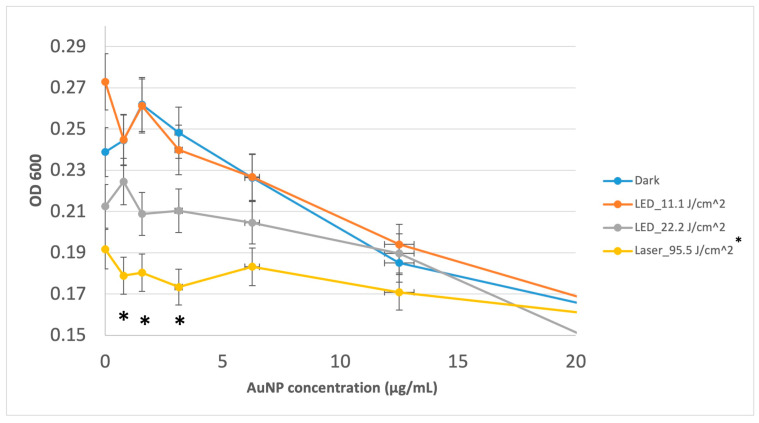
OD600 readings for *S. mutans* inhibitory responses to low AuNP concentration as a function of light treatment. Data are reported as average ± one standard deviation (n = 9). * Indicates significant difference from the dark treatment. Error bars presented in percentages.

The MIC of the gold nanoshells used in this study was observed at 100 µg/mL (for all treatments). The photothermal effect under laser illumination showed a significant reduction in *S. mutans* absorbance at 0.78 µg/mL (*p* < 0.001), 1.56 µg/mL (*p* < 0.001), and 3.12 µg/mL (*p* < 0.001) when compared to the dark treatment (Figure 1). In addition, the laser illumination (95.5 J/cm^2^), when compared to the 15 min LED illumination (11.1 J/cm^2^), showed a significant reduction in *S. mutans* absorbance at 0 µg/mL (*p* < 0.001), 0.78 µg/mL (*p* = 0.005), 1.56 µg/mL (*p* < 0.001), and 3.12 µg/mL (*p* = 0.004). Moreover, the 30 min LED illumination (22.2 J/cm^2^) compared to the dark condition had a significant reduction (*p* = 0.039) at 1.56 µg/mL concentration. The 30 min LED had a significant reduction compared to 15 min LED at 0 µg/mL concentration (*p* = 0.023) (Figure 2)

### 3.3. Colony Forming Unit (CFU) Assay

The CFU test was performed using three different concentrations of gold nanoshells (including the control) and five different light settings.

#### 3.3.1. CFU Assay of SPP_RB Treated with Red LED Light

**Figure 3:**
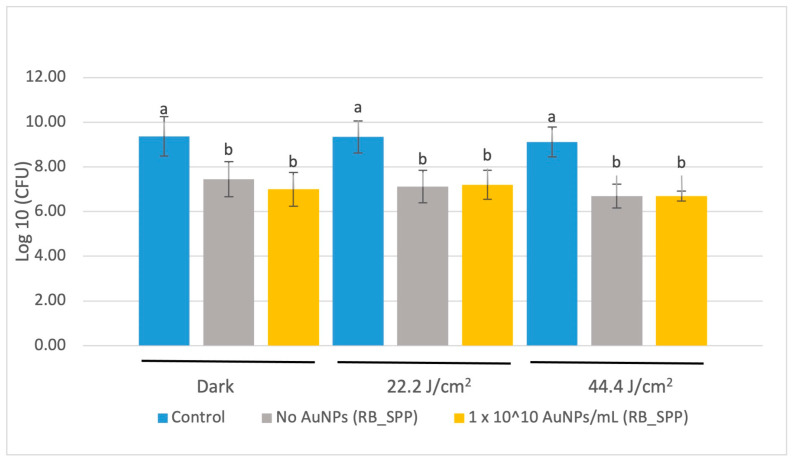
CFU count following application of 675 nm red LEDs. The base resin blend was prepared with Scientific Polymer Products BisEMA (RB_SPP). Data are reported as an average ± one standard deviation (n = 9). Different letters (a, b) indicate statistically different means (*p* < 0.05).

There was no significant difference reported in CFU concentrations for *S. mutans* between the 0% (unloaded) and the 1 × 10^10^ AuNP/mL polymerized resin disk surfaces after red LED light illumination (at a maximum wavelength of 675 nm) for either applied energy studied (Figure 3). The difference observed between the control and the resin disks (both 0% and gold nanoshell included) is due to surface area differences between the resin disk surface and the culture well plate.

#### 3.3.2. CFU Assay of SPP_RB Treated with a Laser

**Figure 4:**
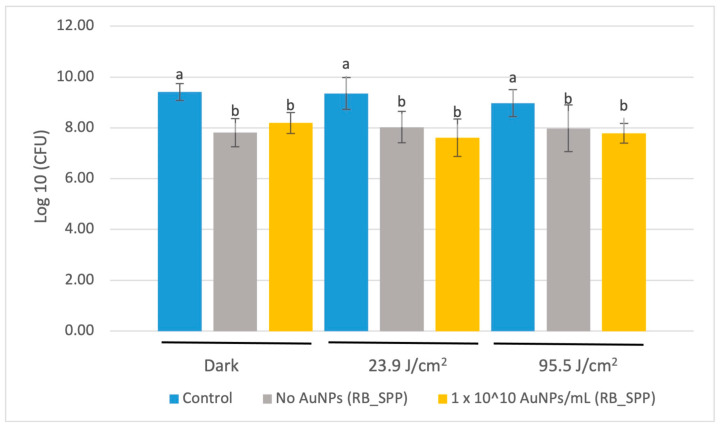
CFU count following application of a 660 nm red laser. The base resin blend was prepared with Scientific Polymer Products BisEMA (RB_SPP). Data are reported as an average ± one standard deviation (n = 9). Different letters (a, b) indicate statistically different means (*p* < 0.05).

There was no significant difference reported in CFU concentration for *S. mutans* between the unloaded and 1 × 10^10^ AuNP/mL polymerized resin disk surfaces after laser illumination (at 660 nm wavelength, 100 mW) for either energy applied (Figure 4).

#### 3.3.3. CFU Assay of EI_RB Treated with Laser

**Figure 5:**
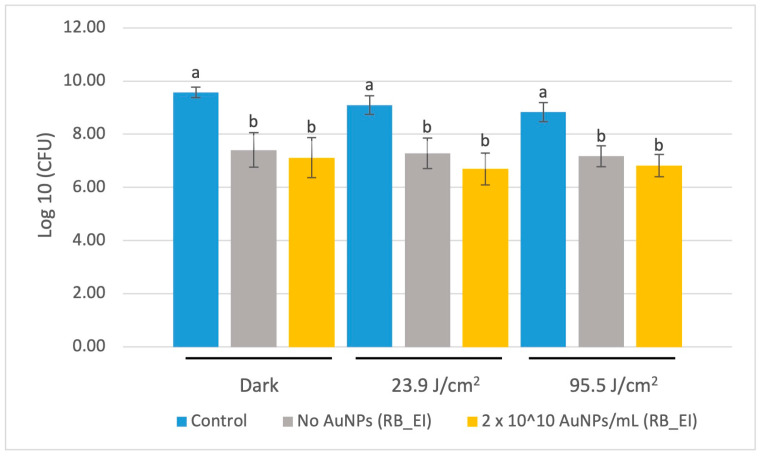
CFU count following application of a 660 nm red laser. The base resin blend was prepared with Esstech Incorporated BisEMA (RB_EI). Data are reported as an average ± one standard deviation (n = 9). Different letters (a, b) indicate statistically different means (*p* < 0.05).

*S. mutans* count reduction was not significant between the 0% (unloaded) and the 2 × 10^10^ AuNP/mL polymerized resin disk surfaces after laser illumination (at 660 nm wavelength, 100 mW, 30 s) for either energy applied (Figure 5).

### 3.4. UV-Vis Analysis

**Figure 6:**
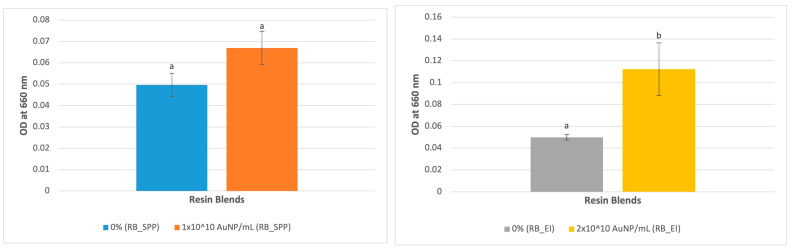
UV-Vis of (**left**): blends made with SPP-sourced BisEMA and (**right**): blends made with EI-sourced BisEMA as a function of AuNP absorbance peak at 660 nm. Data are reported as an average ± one standard deviation (n = 9). Different letters (a, b) indicate statistically different means (*p* < 0.05).

The absorbance for the 2 × 10^10^ AuNP/mL concentration resin blend was significantly higher at 660 nm (peak absorbance for gold nanoshells) (*p* = 0.009) (Figure 6–right), which indicates higher concentration of gold nanoparticles’ presence in the blend. The absorbance for 1 × 10^10^ AuNP/mL concentration was higher although not significant (Figure 6–left).

## 4. Discussion

In our study, gold nanoshells (AuNPs) expressed enhanced antibacterial effects alongside the application of PTT using low concentrations of AuNPs in culture media with planktonic *S. mutans*. Even without the use of PTT, at high AuNP concentrations, there was still some antibacterial effect detected. However, while adding AuNPs to a dental resin blend did not compromise the degree of conversion, the *S. mutans* response did not change as a result of AuNP loading within the photopolymerized dental resin. In addition, while UV-Vis confirmed presence of the AuNPs in the resin blend, SEM-EDX was unable to detect it on the polymerized resin disk surface. As such, AuNPs are believed to be well mixed within the resin and will be covered by resin on the disk surface. The interlocked gold nanoshells, therefore, might not be able to transfer the heat within the dental resin adhesive, which is a non-conductive material to kill or deactivate *S. mutans* on the surface of the polymerized resin disks. Although we observed spherical shapes around the size of the gold nanoshells used in this study on the surface of the resin disks via SEM, EDX did not confirm the presence of gold. Since the EDX detection limit is 1 to 2 microns deep, if the gold particles are covered by the resin blend, it cannot detect them. This confirms the interlocking of gold nanoparticles by the resin blends. Future studies will consider other analyses, such as TEM, to further assess the distribution of the particles within the polymerized samples.

Gold nanoparticles have been successful in enhancing osteointegration in dental implants [33], preventing secondary caries by inhibiting the matrix metalloproteinases in the hybrid layer [6], improving cavity disinfectants by enhancing their antibacterial properties [34], assisting in diagnosis of oral squamous cell carcinoma [35], and presenting some antibacterial effects in a dental adhesive resin blend at certain concentrations without the effect of light or PTT [36]. While most studies of AuNPs in dentistry have utilized them in a solution form [6,35], those that have included them in solid dental materials often perform antibacterial tests without significant statistical findings and/or analysis [36]. Furthermore, these aforementioned studies have been conducted under dark conditions. PTT has shown significant potential to improve the antibacterial properties of PTTA-loaded materials [14]. To our knowledge, this is the first study on the response of *S. mutans* in both AuNP-incorporated resin blends and the application of PTT.

The lack of *S. mutans* response to PTT of the AuNP-loaded resin in this study may be due to the limited heat transfer within the dental resin blend, as well as no detected AuNP on the resin surface. Without sufficient heat transfer to the point of contact with the bacteria, it would not be possible to kill or deactivate *S. mutans.* Nanoparticles were selected here to provide a high surface area for any chemical and physical interactions to occur [37]. To take better advantage of the high surface area of nanoparticles and overcome the limited heat transfer of the polymerized resin, future studies should consider increasing the concentration of AuNPs directly on the material’s surface. For instance, Estrela et al. (2016) suggested that the lack of antibacterial activity was due to the entrapment of silver nanoparticles in the resin luting cement and their limited direct contact with the bacteria [38].

Gold particles come in different sizes and shapes. In 2003, Hirsch et al. were the first to use AuNP nanoshells with a silica core (110 nm particle size) to treat breast carcinomas with a PTT approach [39]. Following that study, different gold particles with various shapes and sizes were introduced for different medical applications [25,35,40,41]. However, there is some conflict in the literature as to whether smaller particles are best for PTT or not. For example, Gupta et al. (2021) suggested that smaller particles are more capable of producing heat, with significant dependence on the medium in which they are loaded [42]. Ayala-Orozco et al. (2014) also recommended that gold nanoshells are still the most suitable nanoparticle due to their ability to sustain absorption, as well as their biocompatibility and low toxicity in the human body [31]. In contrast, Chen et al. (2022) suggested that small nanoparticles can undergo morphology changes under light irradiation, while large nanoparticles will have more stable plasmonic effects [43]. As a result, they introduced plasmonic nanoparticle clusters (PNCs) as the future of PTTAs, proposing that they would be more efficient than small stand-alone particles for PTT [43]. However, Chen et al. (2022) did not evaluate dispersed nanoparticles and the dependence of PTT on nanoparticle size and/or clustering. In our study, gold nanoshells of 120 nm particle size were chosen due to their low toxicity and excellent SPR towards PTT [29,30].

The MIC of AuNPs for *S. mutans* was found to be at 100 μg/mL, with no dependence on light treatment in this study. Previous studies have shown the MIC to be from 25 μg/mL to 120 μg/mL for different sizes and shapes of AuNPs in dark conditions [44,45,46]. In this study, MIC is defined as the inhibition of at least 95% *S. mutans* (OD600), and with a standard deviation in OD600 nm being approximately 5%, the application of PTT should not produce a further detectable inhibition at this same concentration. Meanwhile, for AuNPs concentrations at and below 3.12 μg/mL, the application of 95.5 J/cm^2^ laser light (660 nm) detectably inhibited *S. mutans* by ~25% compared to the dark and LED_11/1 J/cm^2^ light (675 nm) treatments. The AuNPs exhibited antibacterial properties at higher concentrations independent of any light treatment.

The 1 × 10^10^ AuNP/mL concentration was selected for this study based on prior investigation by Pattani et al. (2012) in which a temperature difference of 45 °C in tissue phantoms was reported [30]. Furthermore, after not observing the antibacterial effect expected, we doubled the concentration to 2 × 10^10^ AuNP/mL, which is the maximum concentration possible within the limitations of this study. Moreover, we applied the maximum power delivery method available within the limitations of this study to the surface of the resin disks in the CFU test using an LED device and a laser commonly available in a dental setting. However, it is well recognized that different materials have different thermal conductivities. For example, metallic nanoparticles may be used to increase the thermal conductivity in dental materials, but this often comes with a loss in other properties, such as tensile strength [47,48]. We could not observe a temperature difference during the bacterial tests using an industrial IR thermometer. Due to the limitations of this study, the use of other devices to measure temperature differences would come at the risk of sample contamination if performed out of the biosafety cabinet. Future studies will seek to optimize the thermal conductivity of our experimental resin as a function of AuNP concentration in order to produce a more agonistic antibacterial response. The real question to be answered in future studies is what type of photothermal agent (shape, size, loading, concentration, etc.) best fits the need of the dental biomaterials for different purposes to benefit from their photothermal effect. We chose gold nanoshells based on the literature that exists mostly within the field of medicine and cancer treatments. The lack of studies in dentistry and dental biomaterials towards photothermal therapy is still unknown. As this study is the first to demonstrate the benefits of utilizing AuNPs in a PTT-based approach for managing cariogenic bacteria, these results should encourage further research towards the development of antibacterial dental materials associated with a PTT approach.

## 5. Conclusions

While the application of PTT improved inhibition of planktonic *S. mutans* in low aqueous concentrations of AuNPs, the impact of PTT on the antibacterial properties of AuNP-loaded resin was not similarly observed. The addition of the AuNPs to the dental resin did not compromise the degree of conversion; this potentially offers the opportunity to load an even greater concentration of AuNPs in future investigations. Future studies should further investigate different AuNPs types (size, shape, surface chemistry, loading mode, etc.) and consider higher concentrations in a similar approach to fully understand the potential of this technology. It is possible that a higher concentration of AuNPs loaded into a resin blend may express stronger photothermal effects to reduce *S. mutans*. The results of this study will potentially pioneer the application of photothermal therapy to control oral pathogenic bacteria linked to dental caries.

## Figures and Tables

**Table 1:** Base resin blend formulation.

Material	Weight %
Bisphenol A Dimethacrylate (BisEMA) ^1^	70%
Tetraethylene-glycol-dimethacrylate (TEEGDMA)	26%
Camphorquinone (CQ)	0.66%
Ethyl 4-(dimethylamino) benzoate (Amino)	1.34%
Ethanol	2%

^1^ Due to the discontinuation of BisEMA from Scientific Polymer Products mid-study, this paper presents an investigation using two BisEMAs from different sources. An appropriate unloaded control of matching BisEMA source was utilized in each characterization.

**Table 2:** MIC assay treatments.

Light Device	Time/Distance	Energy Delivery
LED red light ^1^	15 min	11.1 J/cm^2^
LED red light	30 min	22.2 J/cm^2^
Laser ^2^	30 s/2 mm	95.5 J/cm^2^

^1^ Custom in-house LED device. ^2^ SiroLaser Blue, Dentsply Sirona (Charlotte, NC, USA). The tip of the laser was leveled to the surface of the 96-well plate.

**Table 3:** Different light settings used for the CFU test.

Light Device	Time/Distance	Energy Delivery
LED red light ^1^	30 min	22.2 J/cm^2^
LED red light	60 min	44.4 J/cm^2^
Laser ^2^	30 s/2 mm	95.5 J/cm^2^
Laser ^3^	30 s/4 mm	23.9 J/cm^2^

^1^ Custom in-house LED device. ^2^ SiroLaser Blue, Dentsply Sirona (Charlotte, NC, USA). The tip of the laser was marked and leveled to the surface of the 24-well plates to have the correct distance from the surface of the resin disks. ^3^ For 2 × 10^10^ AuNP/mL disks, only the laser illumination was used to deliver maximum energy.

**Table 4:** Degree of conversion as a function of AuNP concentration (and BisEMA source). Data are reported as average ± one standard deviation (n = 6). Different letters indicate statistically different means (*p* < 0.05).

Resin Blend	Degree of Conversion %
0% (RB_SPP)	70.86 ± 5.03 ^a^
1 × 10^10^ AuNP/mL (RB_SPP) ^1^	64.08 ± 3.11 ^ab^
0% (RB_EI)	57.23 ± 5.11 ^b^
2 × 10^10^ AuNP/mL (RB_EI)	60.24 ± 5.05 ^b^

^1^ Two different BisEMAs were used from different companies due to product discontinuation. RB_SPP—Scientific Polymer Products-sourced BisEMA resin blend; RB_EI—Esstech Inc.-sourced BisEMA resin blend.

## Data Availability

The raw data supporting the conclusions of this article will be made available on reasonable request by contacting Dr. Shayan Darvish at sdarvish@umich.edu.

## Supplementary Materials

The following supporting information can be downloaded at: https://www.mdpi.com/article/10.3390/jfb15040100/s1, Figure S1: SEM images of the resin disks surface loaded with 0%, 1 × 10^10^ AuNP/mL, and 2 × 10^10^ AuNP/mL, recorded at 10 kX; Figure S2: EDX results of the surface of the resin disks loaded with 0%, 1 × 10^10^ AuNP/mL, and 2 × 10^10^ AuNP/mL recorded at at 20 kv; Figure S3: Certificate of gold nanoshell analysis by NanoComposix.
