# Supplementary material for: Antibacterial Properties of an Experimental Dental Resin Loaded with Gold Nanoshells for Photothermal Therapy Applications
Source: J Funct Biomater. 2024 Apr 11;15(4):100. doi: 10.3390/jfb15040100 (PMC11051398; doi:10.3390/jfb15040100)
Supplement: Supplementary file 1 [file jfb-15-00100-s001.zip › jfb-2937175-supplementary.pdf]

Journal of *Functional Biomaterials*

Supplementary Materials

Antibacterial Properties of an Experimental Dental Resin Loaded with Gold Nanoshells for Photothermal Therapy Applications

Shayan Darvish [1], Dana-Gabriela Budala [2,*] and Ancuta Goriuc [3]

1 Department of Oral Health Sciences, Faculty of Dentistry, The University of British Columbia, Vancouver, BC V6T 1Z3, Canada; sdarvish@umich.edu
2 Department of Prosthodontics, Faculty of Dental Medicine, "Grigore T. Popa" University of Medicine and Pharmacy, 16 Universității Street, 700115 Iași, Romania
3 Department of Biochemistry, Faculty of Dental Medicine, "Grigore T. Popa" University of Medicine and Pharmacy, 16 Universității Street, 700115 Iasi, Romania; ancuta.goriuc@umfiasi.ro
* Correspondence: dana-gabriela.bosinceanu@umfiasi.ro

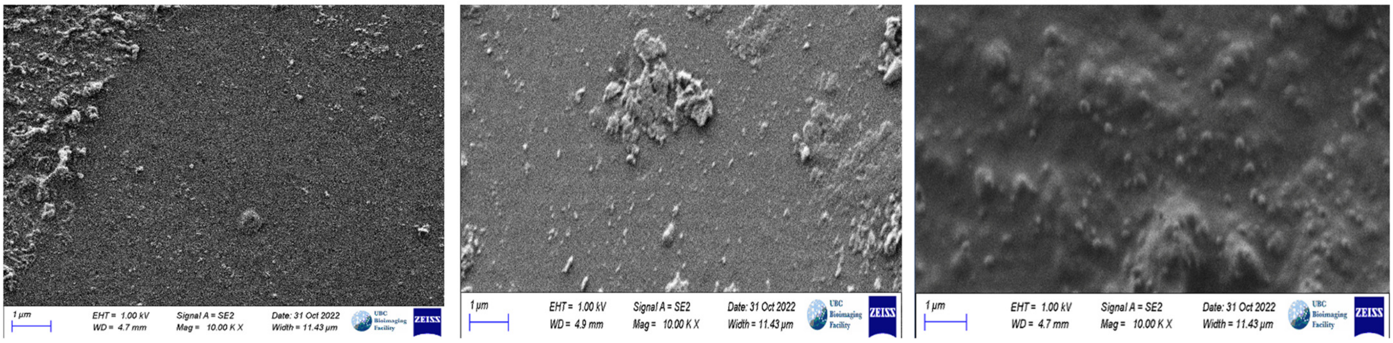

0% | 1×10^{10} AuNP | 2×10^{10} AuNP

Figure S1. SEM images of the resin disks surface loaded with 0%, 1×10^{10} AuNP/ml, and 2×10^{10} AuNP/ml recorded at at 10kX.

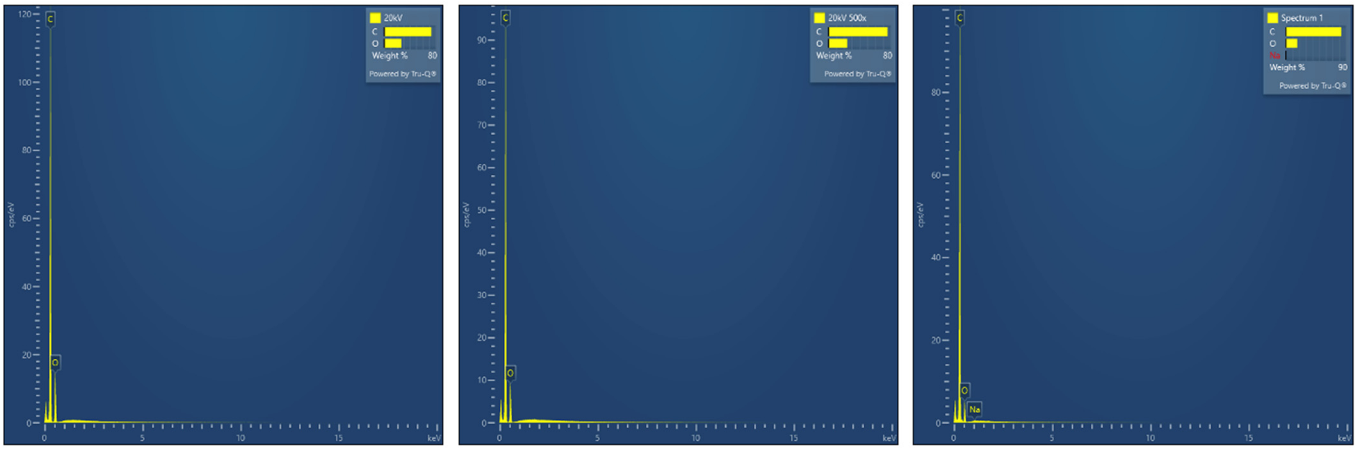

0% | 1×10^{10} AuNP | 2×10^{10} AuNP

Figure S2. EDX results of the surface of the resin disks loaded with 0%, 1×10^{10} AuNP/ml, and 2×10^{10} AuNP/ml recorded at at 20 kv.

nanoComposix **Certificate of Analysis**

Gold Nanoshells, Peak Absorbance @ 660 nm, Lipoic Acid, NanoXact™

Product Number: GSLH660 **Lot Number: WTJ0543**

**Total Diameter ± Std.Dev (TEM)*:**	117 ± 6 nm	**Mass Concentration (Au):**	1.08 mg/mL
**Coefficient of Variation*:**	5.0 %	**Hydrodynamic Diameter:**	129 nm
**Core Diameter ± Std.Dev (TEM)*:**	78 ± 5 nm	**Zeta Potential:**	-62 mV
**Shell Thickness (Calc'd):**	20 nm	**pH of Solution:**	7.4
**Surface Area (Calc'd):**	3.6 m^2/g	**Particle Surface:**	Carboxyl (Lipoic Acid)
**Particle Concentration (Calc'd):**	9.5E+10 particles/mL	**Solvent:**	0.02 mM Potassium Carbonate
**% Absorbance at 660 nm:**	99.3 %		

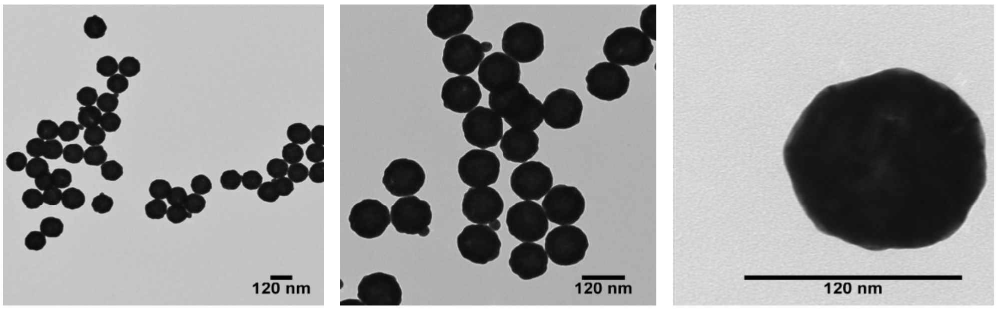

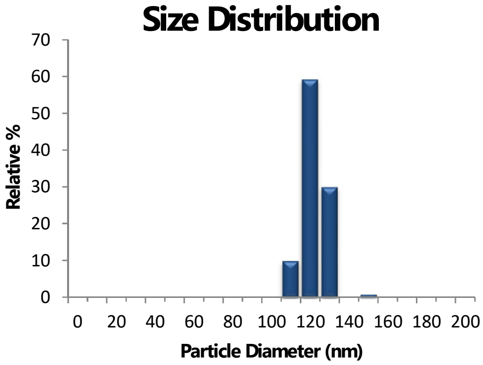

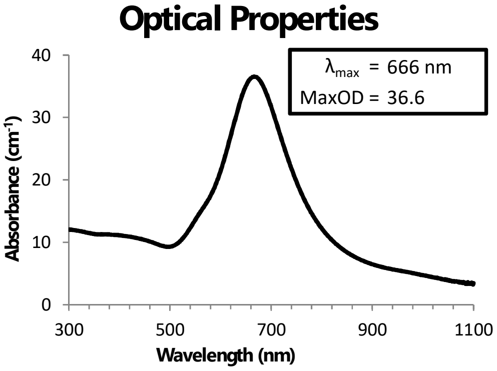

Characterization Instrumentation

**Diameter and Size Statistics:**	JEOL 1010 Transmission Electron Microscope; ImageJ
**Mass Concentration:**	Thermo Fisher X Series 2 ICP-MS
**Spectral Properties:**	Agilent 8453 UV-Visible Spectrometer
**Hydrodynamic Diameter/Zeta Potential:**	Malvern Zetasizer Nano ZS
**pH:**	Horiba - Laqua Twin pH Meter

Shake vigorously before use. Bath sonicate if needed. Storage: 2-8 ^{o}C. DO NOT FREEZE.

*Particle size data is based on measurements of singlet particles only.

nanoComposix, Inc
4878 Ronson Ct. Suite K
San Diego, CA 92111

nanoComposix.com

info@nanocomposix.com
Phone: (858) 565-4227
Fax: (619) 330-2556

Figure S3. Certificate of gold nanoshell analysis by NanoComposix.
